# PR status is a more decisive factor in efficacy of adding pertuzumab into neoadjuvant therapy for HER2-positive and lymph node-positive breast cancer than ER status: a real-world retrospective study in China

**DOI:** 10.1186/s12957-023-03178-4

**Published:** 2023-09-18

**Authors:** Xiaoyu Liu, Zhaoyun Liu, Chao Li, Xiang Song, Xinzhao Wang, Sumei Li, Zhiyong Yu

**Affiliations:** 1 First Clinical Medical College, Shandong University of Traditional Chinese Medicine, Jinan, China; 2 Breast Cancer Center, Shandong Cancer Hospital and Institute, Shandong First Medical University and Shandong Academy of Medical Sciences, Jinan, China; 3 College of Traditional Chinese Medicine, Shandong University of Traditional Chinese Medicine, Jinan, China

**Keywords:** HER2-positive breast cancer, Progesterone receptor, Neoadjuvant therapy, Pathological complete response

## Abstract

**Background:**

Although neoadjuvant trastuzumab and pertuzumab (HP)-based regimens are recommended for human epidermal receptor-positive (HER2 +)/lymph node-positive (N +) breast cancer (BC) patients according to NCCN guidelines, it is undeniable that many patients achieved pathological complete response (pCR) after trastuzumab (H)-based regimens without adding pertuzumab to treatment. Patients who specifically benefit from pertuzumab must be identified. The aim of this retrospective study was to evaluate progesterone receptor (PR) status as a predictor of response to the addition of pertuzumab in HER2 + /N + breast cancer.

**Methods:**

One hundred forty-two patients who were diagnosed as HER2 + /N + BC without distant metastasis and followed by neoadjuvant HP-based or H-based therapy were retrospectively included. The endpoints were pCR and disease-free survival (DFS) times.

**Results:**

In total, the pCR occurred in 25 of 87 patients (28.74%) in group H compared with 32 of 55 (58.18%) in group HP. The results revealed that hormone receptor (HR) status was significantly different on pCR in group HP. The odds of pCR for patients who have HR-positive tumors were 0.160 times (*P* = 0.011) that for patients with HR-negative tumors by multivariable analysis. Moreover, a similar probability of PR-positive (PR +) patients, whatever estrogen receptor (ER) status was, achieving pCR in group HP was observed. The ROC curves showed different anti-HER2 regimens provide worst predictive value in the PR + cohort (N = AUC = 0.521, 95% CI: 0.348–0.694, *P* = 0.813) compared with the overall cohort (AUC = 0.644, 95% CI: 0.550–0.738, *P* = 0.004) and ER + cohort (AUC: 0.559, 95% CI: 0.405–0.713, *P* = 0.451). And PR status (AUC = 0.760, 95% CI: 0.626–0.894, *P* = 0.001) had a greater predictive value than ER status (AUC = 0.658, 95% CI: 0.508–0.807, *P* = 0.048) in group HP. DFS analyses were done on 141 patients. Although ER and PR status did not show significant difference in group HP (*P* = 0.789 and 0.088, respectively), HP-based therapy contributed to better DFS in the ER − and PR − cohorts (*P* = 0.035 and 0.015, respectively).

**Conclusions:**

Compared with ER status, PR status might be a more valuable factor predicting the efficacy of adding pertuzumab into neoadjuvant therapy for HER2 + /N + BC. PR + patients benefit little from the addition of pertuzumab.

**Supplementary Information:**

The online version contains supplementary material available at 10.1186/s12957-023-03178-4.

## Introduction

Breast cancer (BC) is the most common cancer and a leading cause of death among women worldwide [1]. The HER2-positive (HER2 +) subtype occurs in approximately 15–20% of all BC cases [2]. Without HER2-directed treatment, HER2 + BC has an aggressive course of disease and poor prognosis [3]. In HER2 + BC, neoadjuvant therapy is preferred over sequential treatment. Neoadjuvant therapy is an effective approach to allow for subsequent surgery in cases that are initially inoperable or allow for breast-conserving surgery rather than mastectomy. It is also for testing in vivo sensitivity to chemotherapy and has become a widely accepted initial treatment in BC patients with unfavorable tumor characteristics or with axillary lymph node metastasis [4]. Pathological complete response (pCR) is recognized as a valid endpoint for neoadjuvant trials and the basis for drug approval [5]. The achievement of pCR to treatment is a representative marker of improved long-term outcomes, especially in HER2 + BC [6].

Once upon a time, trastuzumab (H)-based neoadjuvant therapy was the standard of care in HER2 + BC. However, a significantly higher proportion of patients given neoadjuvant trastuzumab and pertuzumab (HP)-based regimens achieved pCR than those given H-based regimens set the precedent for the routine use of dual HER2-targeted neoadjuvant therapy [7]. HP-based neoadjuvant therapy is recommended to patients with HER2 + and a high risk of recurrence, which is defined as primary tumors measuring more than 2 cm, or node-positive (N +) disease, according to NCCN guidelines [8].

Hormone receptors (HR) status is an important factor affecting pCR in HER2 + patients. Significantly lower pCR rates of HR-positive (HR +) tumors are observed in HER2 + BC compared with HR-negative (HR −) tumors. Besides, pCR rates of HR + patients do not differ much between HP-based and H-based treatment [9]. Despite that estrogen receptor (ER) status was reported as a predictive marker within HER2 + BC that affects disease outcomes [10], the progesterone receptor (PR) status has not been studied individually before. Additionally, N + patients are candidates for neoadjuvant chemotherapy and dual HER2-targeted therapy [8], but there is insufficient evidence that all N + patients benefit from adding pertuzumab into therapy.

Therefore, our study focused on HER2 + /N + breast cancer patients with the goal of pCR and DFS times to evaluate PR status as a predictor of response to neoadjuvant trastuzumab plus pertuzumab treatment in HER2 + /N + breast cancer.

## Methods

### Patient populations

The study included 142 female patients with HER2 + invasive BC treated with H-based or HP-based anti-HER2 neoadjuvant treatment with standard chemotherapy between January 2017 and November 2021. All the patients had primary tumors measuring more than 2 cm and metastatic lymph node(s) without distant metastasis and a history of breast or systemic cancer. Following neoadjuvant therapy, patients underwent surgery and continued further comprehensive treatment.

### Assessments

The invasive breast tumor with node metastasis was confirmed clinically, on imaging, or on cytology/histopathology. Tumors had to be verified as HER2 + by immunohistochemistry (IHC) and fluorescence in situ hybridization (FISH). ER positivity and PR positivity were defined as at least 1% of nuclear staining in tumor cells. And HR + was defined as ER and/or PR positivity. The age of patients, the menopausal status, the histopathological features (tumor size, axillary lymph node involvement, ER status, PR status, HER2 status, Ki-67 index), and comprehensive treatments after surgery were extracted from electronic medical records and pathological records.

The primary study endpoint was pCR. No evidence of a residual invasive tumor in the breast and axillary lymph nodes (ypT0N0/ypTisN0) upon surgical resection was considered to be pCR. And the secondary endpoint was disease-free survival (DFS). DFS was defined as the duration of time from surgical resection to the date of occurring recurrence or distant metastasis. The last follow-up was conducted in July 2023.

### Group and cohort definition

Patients treated with H-based treatment were enrolled in group H, while patients treated with HP-based treatment were enrolled in group HP. All patients in total were defined as overall the cohort. Among them, PR-positive (PR +) patients were defined as the PR + cohort, and PR-negative (PR −) patients were defined as the PR − cohort. ER-positive (ER +) patients were defined as the ER + cohort, and ER-negative (ER −) patients were defined as the ER − cohort.

### Statistical analysis

The GraphPad Prism 9.3.1 software was used in our study for statistical analysis. The differences of pCR rates among groups were shown with percentage and standard errors. Categorical variables were expressed as proportions or odds ratio (OR) and analyzed using the chi-square test or Fisher’s exact test. Univariate and multivariate logistic models were used to determine potentially important prognostic factors for the entire cohort with Exp (B) and 95% confidence interval (CI). Receiver operating characteristic (ROC) curves were applied to represent the predictive value of the factors, corresponding area under the ROC curve (AUC). DFS analysis for each group or cohort was determined using the Kaplan–Meier survival, log-rank test, and Cox regression model estimate. A two-sided *P*-value of 0.05 or less was characterized as statistically significant.

## Results

### Patient baseline characteristics and treatment administration

In total, 142 patients were included, with 87 patients in group H and 55 patients in group HP. Baseline characteristics were balanced between group H and group HP (*P* > 0.05), as shown in Table 1.

**Table 1:** Patient baseline characteristics

Characteristics	Overall cohort (*N* = 142)		
	Group H *N* = 87 (%)	Group HP *N* = 55 (%)	*P*-value
Age			
≤ 50	47 (54.02)	31 (56.36)	0.699
> 50	40 (45.98)	24 (43.64)	
Menopausal status			
Premenopausal	49 (56.32)	32 (58.18)	0.758
Postmenopausal	38 (43.68)	23 (41.82)	
cT stage (pre-treatment)			
1	7 (8.05)	5 (9.09)	0.218
2	54 (62.07)	39 (70.91)	
3	13 (14.94)	4 (7.27)	
4	13 (14.94)	7 (12.73)	
HR			
Negative	38 (43.68)	26 (47.27)	0.553
Positive	49 (56.32)	29 (52.73)	
Her2			
IHC 2 + /FISH +	13 (14.94)	5 (9.4)	0.149
IHC 3 +	74 (85.06)	50 (90.6)	
Ki67			
< 20%	9 (10.34)	7 (12.5)	0.536
≥ 20%	78 (89.66)	48 (87.5)	

All preoperative and 141 postoperative treatments were analyzed (one patient in group H was lost to follow-up). Most patients experienced 4 cycles of HER2-targeted therapy (55.17% of group H and 52.73% of group HP). A chemotherapy regimen containing anthracycline, cyclophosphamide, and taxanes is the most common regimens in the neoadjuvant setting (71.26% of group H and 65.45% of group HP). After surgery, all patients accepted comprehensive treatment for their condition, including targeted therapy, radiotherapy, chemotherapy, and endocrinotherapy. Detailed information is shown in Table 2.

**Table 2:** Treatment administration

Treatment administration	Group H	Group HP	*P*-value
Targeted therapy cycles before surgery	*N* = 87 (%)	*N* = 55 (%)	
4	48 (55.17)	29 (52.73)	0.913
6	24 (27.59)	17 (30.91)	
Others	15 (17.24)	9 (16.36)	
Chemotherapy regimens before surgery	*N* = 87 (%)	*N* = 55 (%)	
AC-T	62 (71.26)	36 (65.45)	0.754
TCb	19 (21.84)	14 (25.45)	
T	6 (6.90)	5 (9.09)	
Breast surgery	*N* = 87 (%)	*N* = 55 (%)	
Breast-conserving surgery	3 (3.45)	5 (9.09)	0.261
Mastectomy	84 (96.55)	50 (90.91)	
Lymph node surgery	*N* = 87 (%)	*N* = 55 (%)	
Sentinel node biopsy	24 (27.59)	17 (30.91)	0.670
Lymph node dissection	63 (72.41)	38 (69.09)	
Treatment after surgery	*N* = 86 (%)	*N* = 55 (%)	
Che + Targ + Rad	27 (31.40)	16 (29.09)	0.886
Endo + Targ + Rad	23 (26.74)	13 (23.64)	
Che + Endo + Targ + Rad	26 (30.23)	16 (29.09)	
Targ + Rad	9 (10.47)	8 (14.55)	
Other	1 (1.16)	2 (3.64)	

*A* Anthracyclines, *C* Cyclophosphamide, *T* Taxanes, *Cb* Carboplatin, *Che* Chemotherapy, *Endo* Endocrinotherapy, *Targ* HER2-targeted therapy, *Rad* Radiotherapy

### PR status has a noticeable effect on pCR for patients who received HP-based regimens

A pCR occurred in 25 of 87 (28.74%) patients in group H compared with 32 of 55 (58.18%) patients in group HP (Table 3). The results revealed that HR status was significantly different on pCR in group HP, while no characteristics showed significant outcomes in group H (Table 4). The pCR rate of patients with HR − breast tumors (OR = 6.491) was significantly higher than that of HR + patients (OR = 0.154) (*P* = 0.002). Similar results were also shown in the analysis of patients achieved ypT0/Tis and ypN0 (Supplementary Table 1). Besides, although the rate of pCR was notably higher for patients with smaller tumors (cT1–3) than those with cT4 tumors in group HP (Exp (B) = 4.167, 95% CI:0.731–23.759), there was no significant difference between the two cohorts (*P* = 0.108 in univariable analysis and *P* = 0.116 in Fisher’s exact test) (Table 3 and Supplementary Table 2). Other interesting results, but with no significance (*P* = 0.104), occurred wherein patients with HER2 IHC 2 + /FISH + tumors (OR = 0.153) had worse response to HP-based therapy than patients with HER2 IHC3 + tumors (OR = 6.526).

**Table 3:** Characteristics of patients achieved pCR

Characteristics	Group H			Group HP				
	Univariable regression			Univariable regression			Multivariable regression	
	*N* (OR)	Exp (B) (95%CI)	*P*-value	*N* (OR)	Exp (B) (95%CI)	*P*-value	Exp (B) (95%CI)	*P*-value
Total	25 (0.346)			32 (2.894)				
Age								
≤ 50	14 (1.118)	1.253 (0.574–2.735)	0.872	19 (1.340)	1.283 (0.422–3.896)	0.660	N/A^a^	N/A
> 50	11 (0.894)	Reference		13 (0.746)	Reference			
Menopausal status								
Pre	15 (1.235)	1.191 (0.462–3.069)	0.717	18 (0.827)	0.827 (0.278–2.459)	0.732	N/A	N/A
Post	10 (0.810)	Reference		14 (1.210)	Reference			
cT stage (pre-treatment)								
1	3 (1.364)	2.250 (0.308–16.411)	0.577	5 (N/A)	4.167 (0.731–23.759)	0.108	1.823 (0.280–11.849)	0.530
2	17 (3.101)	4.125 (0.493–34.499)		22 (0.776)				
3	2 (0.403)	1.632 (0.328–8.112)		3 (2.273)				
4	3 (0.709)	Reference		2 (0.240)	Reference^b^		Reference^b^	
HR								
Negative	12 (1.278)	1.243 (0.488–3.183)	0.649	21 (6.491)	6.873 (2.008–23.552)	0.002	5.097 (1.335–19.465)	0.017
Positive	13 (0.782)	Reference		11 (0.154)	Reference		Reference	
HER2								
IHC2 + /FISH +	4 (1.122)	1.101 (0.305–3.967)	0.884	1 (0.153)	0.153 (0.016–1.475)	0.104	0.336 (0.032–3.530)	0.364
IHC3 +	21 (0.892)	Reference		31 (6.526)	Reference		Reference	
Ki67								
< 20%	2 (0.683)	0.671 (0.129–3.477)	0.634	4 (0.952)	0.952 (0.192–4.732)	0.952	N/A	N/A
≥ 20%	23 (1.464)	Reference		28 (1.050)	Reference			
Chemotherapy regimens before surgery								
AC-T	17 (1.181)	Reference	0.661	21 (1.018)	0.778 (0.217–2.793)	0.650	N/A	N/A
TCb	7 (1.620)	1.511 (0.157–14.499)		9 (1.409)	2.1 (0.312–14.152)			
T	1 (0.475)	0.648 (0.219–1.919)		2 (0.444)	Reference			

^a^*N/A* Not appliable

^b^Setting cT4 as the reference, then compare with cT1-3

**Table 4:** Probability of patients with different ER and PR statuses achieving pCR in overall cohort

PR status	ER status	Group H *N* (%)	Group HP *N* (%)
PR-positive	ER-positive	7 (25.00)	4 (25.00)
	ER-negative	1 (33.33)	3 (37.50)
PR-negative	ER-positive	5 (27.78)	4 (80.00)
	ER-negative	12 (31.58)	21 (80.77)
Total		25 (28.7)	32 (58.2%)

We next constructed a multivariable model with cT stage, PR status, and HER2 status in group HP as covariates for pCR. After adjusting, the odds of pCR for patients who have HR − tumors were 5.097 times (95% CI: 1.335–19.465, *P* = 0.017) that for patients with HR + tumors.

Thus, it triggered speculation that HR status plays an important role in response to the addition of pertuzumab into neoadjuvant therapy.

**Fig. 1:**
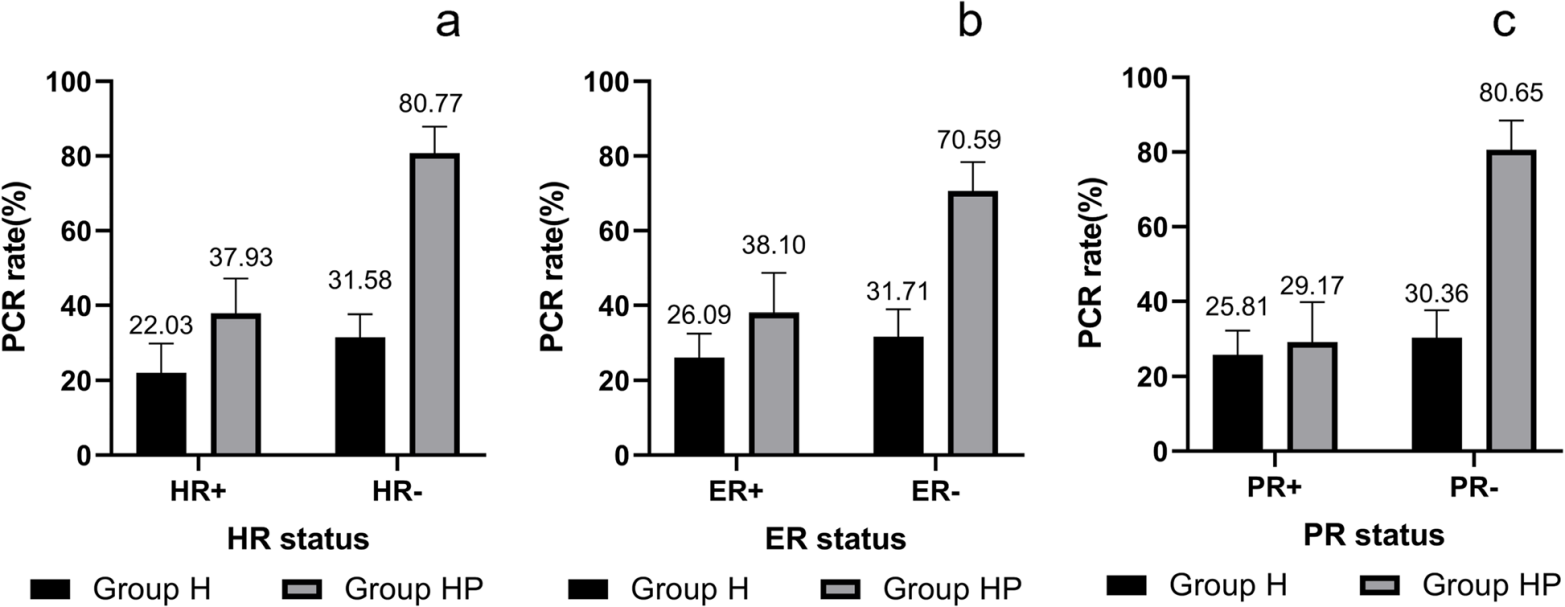
Probability of patients achieving pCR in overall cohort. **a** HR status. **b** ER status. **c** PR status

On the basis of these, the effectiveness of HR status (HR + VS. HR −), ER status (ER + VS. ER −), and PR status (PR + VS. PR −) on pCR was analyzed, respectively. Interestingly, PR + patients had similar pCR rates between group H (25.81%) and group HP (29.17%), while HR + and ER + patients of group HP had higher pCR rates than those of group H (Fig. 1). And the results of univariate analysis showed that in group HP, the odds of pCR for patients who have PR − tumors were 9.212 times (95% CI: 3.327–25.507, *P* < 0.001) that for patients with PR + tumors (Supplementary Table 3). Next, we analyzed the probability of patients with different ER and PR statuses achieving pCR in the overall cohort (Table 4). The probability of PR − patients in group HP achieving pCR was higher than those of in group H, whatever ER status was (ER + : 27.78% of group H vs 80.00% of group HP; ER − : 31.58% of group H vs 80.77% of group HP). However, PR + patients had similar pCR rates between two treatment groups (PR + : 25.00% of group H vs 25.00% of group HP; PR − : 33.33% of group H vs 37.50% of group HP).

Moreover, we constructed a univariable model of patients treated with different chemotherapy regimens in group HP. It revealed that HR (*P* = 0.008), ER (*P* = 0.033), and PR (*P* = 0.004) statuses were significantly different on pCR of patients treated with AC-T in group HP (Supplementary Tables 4 and 5).

**Fig. 2:**
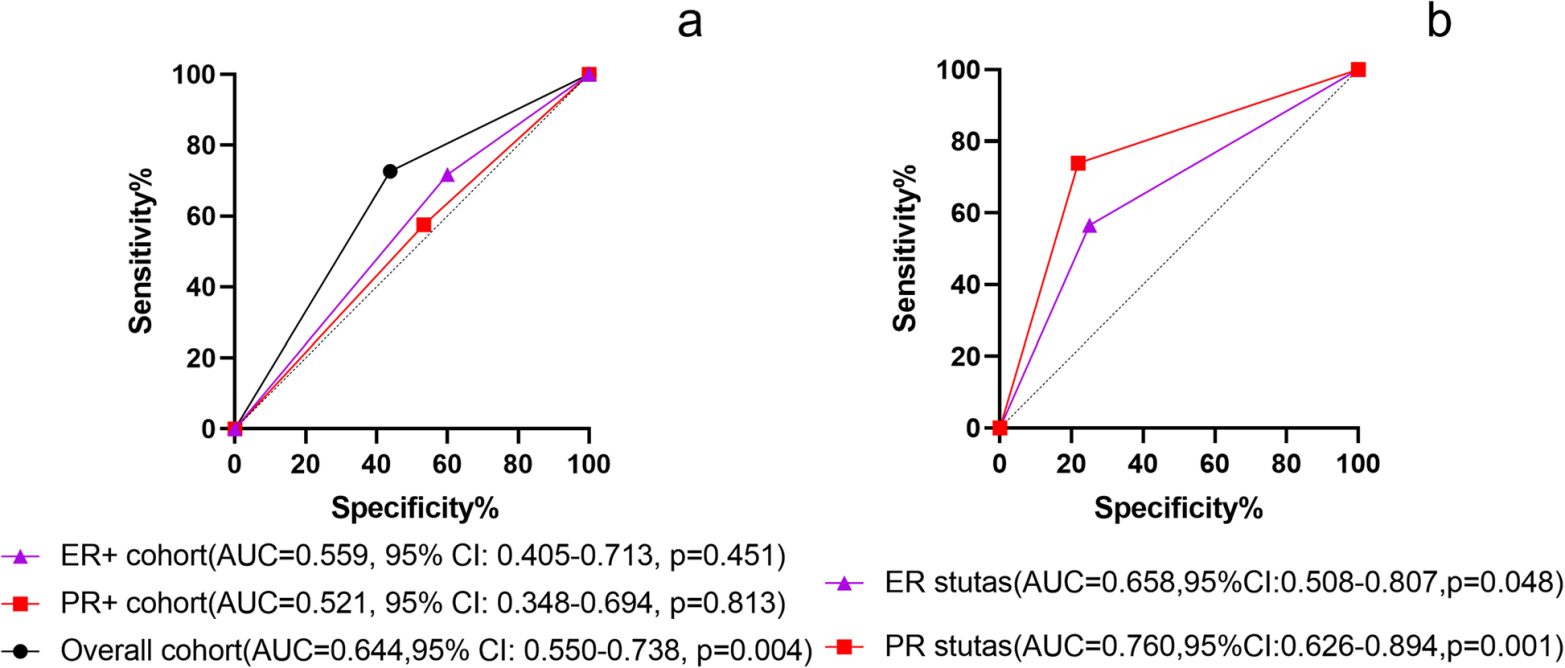
The ROC curves. **a** The ROC curve of anti-HER2 therapy on predicting pCR in different cohorts. **b** The ROC curve of ER and PR status on predicting pCR in group HP

Sixty-six ER + patients (ER + cohort) and 55 PR + patients (PR + cohort) were screened respectively. Of these, 45 patients in the ER + cohort and 31 patients in the PR + cohort belonged to group H. Twenty-one patients in the ER + cohort and 24 patients in the PR + cohort belonged to group HP. Shown in Fig. 2a are the statistically significant impacts of anti-HER2 therapy on pCR in the overall, ER + , and PR + cohorts. The results indicated that the AUC for the overall cohort was 0.644 (95% CI: 0.550–0.738, *P* = 0.004). However, the ER + cohort yielded a smaller area under the ROC curve (AUC = 0.559, 95% CI: 0.405–0.713, *P* = 0.451). And the area under the curve value of PR + cohort was the smallest (AUC = 0.521, 95% CI: 0.348–0.694, *P* = 0.813), suggesting that different anti-HER2 regimens (trastuzumab or trastuzumab and pertuzumab) provide worst predictive value in the PR + cohort. Then, we analyzed the impacts of ER and PR status on pCR in group HP. As shown in Fig. 2b, the area under the ROC curve of the ER and PR status was 0.658 (95% CI: 0.508–0.807, *P* = 0.048) and 0.760 (95% CI: 0.626–0.894, *P* = 0.001), respectively.

Therefore, PR status has a noticeable effect on pCR for patients who received HP-based regimens. PR + patients might benefit less from the addition of pertuzumab than PR − patients.

### *The addition of pertuzumab had low impact on DFS for PR* + *patients*

One patient of group H with non-pCR was lost to follow-up. One hundred forty-one patients were evaluable for DFS analysis. The median follow-up time of group H was 25 (interquartile range 19 to 35.25) months, and that of group HP was 19 (interquartile range 16 to 24) months.

ER + , ER − , PR + , and PR − patients of group H had a median DFS of 21.5 (18.0–31.0), 19.0 (13.8–27.8), 22.0 (19.0–31.0), and 20.0 (14.0–30.0) months, respectively. However, ER + and PR + patients of group HP had shorter DFS times (17.0 (14.8–20.0) and 17.5 (14.3–23.8) months, respectively) than ER − and PR − patients (20.0 (16.0–25.0) and 18.5 (16.0–23.3) months, respectively).

**Fig. 3:**
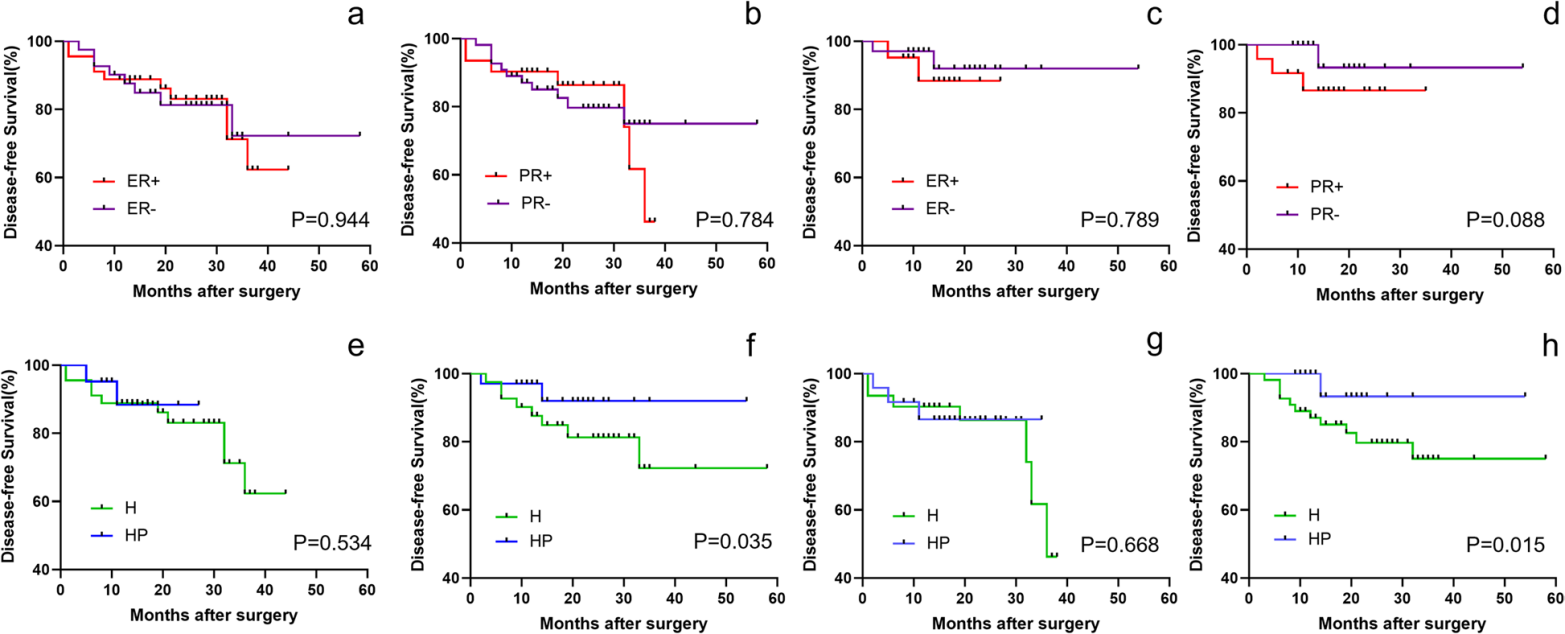
Kaplan–Meier survival curves. **a** Patients with different ER status in group H. **b** Patients with different PR status in group H. **c** Patients with different ER status in group HP. **d** Patients with different PR status in group HP. **e** Patients stratified by anti-HER2 treatment in ER + cohort. **f** Patients stratified by anti-HER2 treatment in ER − cohort. **g** Patients stratified by anti-HER2 treatment in PR + cohort. **h** Patients stratified by anti-HER2 treatment in PR − cohort

No significant difference in the Cox regression model of DFS times of patients with different ER (*P* = 0.944, Fig. 3a) and PR status (*P* = 0.784, Fig. 3b) was observed in group H. Yet, PR status was sightly correlated with DFS (*P* = 0.088, Fig. 3c), while ER status had no significant correlation with DFS (*P* = 0.789, Fig. 3d) in group HP. Moreover, the anti-HER2 treatment was a predictor of DFS times in ER − (*P* = 0.035, Fig. 3e) and PR − cohorts (*P* = 0.015, Fig. 3f) rather than ER + (*P* = 0.534, Fig. 3g) and PR + cohorts (*P* = 0.668, Fig. 3h).

These results suggested that ER − and/or PR − patients treated with HP-based therapy had better long-term outcomes than that treated with H-based therapy, while a different HER2 target therapy (with or without pertuzumab) had low impact on DFS for ER + and /or PR + patients, especially for PR + patients.

Although we analyzed the impact of PR status on different chemotherapy regimens in group HP, the small number of patients resulted in the limitation of analysis, and there was no significant difference in the DFS times of patients treated with AC-T regimen with different PR status (*P* = 0.309) in group HP (Supplementary Figure).

## Discussion

Considering growing concerns with neoadjuvant therapy in HER2 + BC, we here made a retrospective analysis to evaluate PR status as a predictor of response to neoadjuvant dual anti-HER2 therapy in HER2 + /N + breast cancer and provide real-world data to further explore pertuzumab-containing neoadjuvant treatment patterns.

Significantly lower pCR rates of HR + patients than that of HR − patients were wildly reported in the past decades [7, 9, 11]. ER status, known as an important factor of HR, is crucial for the efficacy of anti-HER2 therapy [12]. Although estrogens are the main mitogens in BC, progesterone through its receptor can directly modulate the actions of ER [13]. It has been shown that progesterone signaling modulates BC and the tumor microenvironment [14]. Zhao et al. [15] reported that HER2 + /ER + patients with high PR expression did not benefit from trastuzumab as much as those with low PR expression or PR − . However, PR status has been mostly overlooked. Thus, we hypothesized PR status might be a more decisive factor in the efficacy of adding pertuzumab into neoadjuvant therapy for HER2 + /N + BC than ER status.

The mechanism of pertuzumab and the crosstalk among PR, HER2, and HER3 complicate the interpretations of our findings. As reported, pertuzumab blocks HER dimerization, by binding the dimerization domain of the HER2 receptor (ECD II), and also prevents ligand-induced dimerization of HER2 with HER3 inhibiting tumor growth [16–18]. Hence, the expression of HER2 and HER3 influences the efficacy of pertuzumab. A better response to pertuzumab was achieved in patients with high expression of HER2 and HER3 to a certain extent [19, 20]. However, PR positivity is inversely correlated with HER2 or HER3 expression. Relatively lower levels of HER2 and HER3 in PR + cancer led to a worse response to pertuzumab [21–23]. Furthermore, the presence of functional HER2, HER3, and HER2/HER3 heterodimers affects heregulin-induced transcriptional activation of PR [24]. Also, recurrent activation of PR signaling may promote breast carcinogenesis [25]. Nonetheless, the interactions between PR expression and effectiveness of pertuzumab are still unclear.

Besides, it was reported the pCR rate correlated with the HER2 IHC score in neoadjuvant anti-HER2 treatment [26–30]. And there is a large cohort of T4 patients in the trastuzumab era which showed that distant recurrence-free survival did not differ significantly between non-inflammatory and inflammatory BC for HER2 + tumors [31]. Although patients with a large breast tumor burden or inflammatory (cT4) and IHC 2 + /FISH + patients were reported similar pCR rates between the two treatment groups in our study, additional treatment, including but are not limited to pertuzumab, still should be considered for cT4 and IHC 2 + /FISH-positive BC patients.

In view of the little benefit of HP-based therapy for PR + patients, escalation therapeutic options could be considered for these patients. Some novel drugs, such as small-molecule irreversible tyrosine kinase inhibitor (TKI), antibody–drug conjugates (ADC), and CDK4/6 inhibitor, might provide a pleasing effect for them. Pyrotinib, a commonly used pan-epidermal growth factor receptor TKI, has recently been shown to be clinically effective for the treatment of HER2 + BC in neoadjuvant settings [32–34]. Although the KRISTINE trial results have not changed the standard of care for the neoadjuvant management of HER2 + BC [35], it was reported that a 71% pCR rate was achieved by docetaxel + carboplatin + trastuzumab + pertuzumab (4 cycles) followed by trastuzumab emtansine (T-DM1) + pertuzumab (4 cycles) regimen [36]. Furthermore, neoadjuvant pyrotinib and letrozole plus dalpiciclib showed a promising pathological response in patients with ER + /PR + /HER2 + BC [37].

This retrospective study used a single site in China limiting the number of patients and analysis power. And the scarcity of long-term evidence is noticeable. Although expression of PR has been previously shown to be a strong prognostic factor in survival [38, 39], the real predictive value of PR status on the efficacy of adding pertuzumab into neoadjuvant therapy might be limited. Therefore, a larger sample size and multicenter study are needed to verify the validity and practicability of these outcomes.

### Supplementary Information

**Additional file 1:** **Supplementary Table 1.** Characteristics of Patients Achieved ypT0/Tis and ypN0.

**Additional file 2:** **Supplementary Table 2.** Analysis of patients with small tumors achieved pCR.

**Additional file 3:** **Supplementary Table 3.** The univariate analysis.

**Additional file 4:** **Supplementary Table 4.** Patient baseline characteristics in group HP.

**Additional file 5:** **Supplementary Table 5.** Characteristics of patients achieved pCR in group HP.

**Additional file 6:** **Supplymentary Figure. **a.Patients with different chemotherapy regimens in group HP; b. Patients treated with AC-T regimen with different PR status in group HP.

## Data Availability

The datasets used and/or analyzed during the current study are available from the corresponding author on reasonable request.

## Abbreviations

H: Trastuzumab
HP: Trastuzumab and pertuzumab
N: Lymph node
BC: Breast cancer
pCR: Pathological complete response
ER: Estrogen receptor
PR: Progesterone receptor
HR: Hormone receptors
HER2: Human epidermal growth factor receptor-2
DFS: Disease-free survival
IHC: Immunohistochemistry
FISH: Fluorescence in situ hybridization
OR: Odds ratio
CI: Confidence interval
ROC: Receiver operating characteristic
AUC: Area under the ROC curve
