# Supplementary material for: PR status is a more decisive factor in efficacy of adding pertuzumab into neoadjuvant therapy for HER2-positive and lymph node-positive breast cancer than ER status: a real-world retrospective study in China
Source: World J Surg Oncol. 2023 Sep 18;21:296. doi: 10.1186/s12957-023-03178-4 (PMC10506239; doi:10.1186/s12957-023-03178-4)
Supplement: Supplementary file 1 — Additional file 1: Supplementary Table 1. Characteristics of Patients Achieved ypT0/Tis and ypN0. [file 12957_2023_3178_MOESM1_ESM.docx]

**Supplementary Table 1** Characteristics of Patients Achieved ypT0/Tis and ypN0

| Characteristics | | Chi-square test or Fisher’s exact test | | | | | | | |
| --- | --- | --- | --- | --- | --- | --- | --- | --- | --- |
|  | | ypT0/Tis  N=69 | | | | ypN0  N=83 | | | |
|  | | Group H | | Group HP | | Group H | | Group HP | |
|  | | N(OR) | P-value | N(OR) | P-value | N(OR) | P-value | N(OR) | P-value |
| Total | | 31(0.248) |  | 38(4.038) |  | 47(0.620) |  | 36(1.1613) |  |
| Age | | | | | | | | | |
|  | ≤50 | 18(1.289) | 0.574 | 23(1.725) | 0.352 | 29(1.969) | 0.119 | 21(1.260) | 0.685 |
|  | ＞50 | 13(0.776) |  | 15(0.580) |  | 18(0.508) |  | 15(0.794) |  |
| Menopausal status | | | | | | | | | |
|  | Pre | 17(0.911) | 0.836 | 22(0.963) | 0.949 | 32(2.886) | 0.011 | 20(0.729) | 0.587 |
|  | Post | 14(1.098) |  | 16(1.039) |  | 15(0.306) |  | 16(1.371) |  |
| cT stage (pre-treatment) | | | | | | | | | |
|  | 1 | 4(2.617) | 0.327 | 5(N/A) | 0.094 | 6(5.707) | 0.251 | 5(N/A) | 0.351 |
|  | 2 | 21(1.464) |  | 26(0.667) |  | 29(0.967) |  | 24(0.533) |  |
|  | 3 | 3(0.493) |  | 4(N/A) |  | 7(0.992) |  | 3(1.636) |  |
|  | 4 | 3(0.493) |  | 3(0.279) |  | 5(0.544) |  | 4(0.667) |  |
| HR | | | | | | | | | |
|  | Negative | 13(0.896) | 0.807 | 23(7.156) | 0.004 | 20(0.905) | 0.819 | 22(6.111) | 0.010 |
|  | Positive | 18(1.117) |  | 15(0.140) |  | 27(1.105) |  | 14(0.164) |  |
| HER2 | | | | | | | | | |
|  | IHC 2+/FISH+ | 5(1.154) | 0.817 | 2(0.704) | 0.165 | 7(0.992) | 0.989 | 2(0.314) | 0.327 |
|  | IHC 3+ | 26(0.867) |  | 36(1.421) |  | 40(1.008) |  | 34(3.188) |  |
| Ki-67 | | | | | | | | | |
|  | <20% | 3(0.893) | 1.000 | 5(1.136) | 1.000 | 4(0.686) | 0.729 | 4(0.667) | 0.682 |
|  | ≥20% | 28(1.120) |  | 33(0.880) |  | 42(1.458) |  | 32(1.500) |  |
| Chemotherapy regimens before surgery | | | | | | | | | |
|  | AC-T | 22(0.978) | 0.987 | 26(1.517) | 0.336 | 38(2.815) | 0.056 | 25(1.653) | 0.429 |
|  | TCb | 7(1.069) |  | 10(1.161) |  | 8(0.541) |  | 9(0.933) |  |
|  | T | 2(0.897) |  | 2(0.259) |  | 1(0.152) |  | 2(0.314) |  |
