# Supplementary material for: PR status is a more decisive factor in efficacy of adding pertuzumab into neoadjuvant therapy for HER2-positive and lymph node-positive breast cancer than ER status: a real-world retrospective study in China
Source: World J Surg Oncol. 2023 Sep 18;21:296. doi: 10.1186/s12957-023-03178-4 (PMC10506239; doi:10.1186/s12957-023-03178-4)
Supplement: Supplementary file 2 — Additional file 2: Supplementary Table 2. Analysis of patients with small tumors achieved pCR. [file 12957_2023_3178_MOESM2_ESM.docx]

**Supplementary Table 2** Analysis of patients with small tumors achieved pCR

| cT stage (pre-treatment) | Group H | | Group HP | |
| --- | --- | --- | --- | --- |
|  | N (OR) | P-value | N(OR) | P-value |
| 1-3 | 22(1.410) | 0.749 | 30(4.167) | 0.116 |
| 4 | 3(0.709) |  | 2(0.240) |  |
