# Supplementary material for: PR status is a more decisive factor in efficacy of adding pertuzumab into neoadjuvant therapy for HER2-positive and lymph node-positive breast cancer than ER status: a real-world retrospective study in China
Source: World J Surg Oncol. 2023 Sep 18;21:296. doi: 10.1186/s12957-023-03178-4 (PMC10506239; doi:10.1186/s12957-023-03178-4)
Supplement: Supplementary file 3 — Additional file 3: Supplementary Table 3. The univariate analysis. [file 12957_2023_3178_MOESM3_ESM.docx]

**Supplementary Table 3** The univariate analysis

|  | Group H | | Group HP | |
| --- | --- | --- | --- | --- |
|  | OR(95%CI) | P-value | OR(95%CI) | P-value |
| ER status | 1.315(0.588-2.945) | 0.505 | 3.323(1.306-8.453) | 0.012 |
| PR status | 1.239(0.515-2.984) | 0.632 | 9.212(3.327-25.507) | ＜0.001 |
| HR status | 1.253(0.574-2.735)) | 0.572 | 5.382(2.009-14.419) | 0.001 |
