# Supplementary material for: PR status is a more decisive factor in efficacy of adding pertuzumab into neoadjuvant therapy for HER2-positive and lymph node-positive breast cancer than ER status: a real-world retrospective study in China
Source: World J Surg Oncol. 2023 Sep 18;21:296. doi: 10.1186/s12957-023-03178-4 (PMC10506239; doi:10.1186/s12957-023-03178-4)
Supplement: Supplementary file 4 — Additional file 4: Supplementary Table 4. Patient baseline characteristics in group HP. [file 12957_2023_3178_MOESM4_ESM.docx]

**Supplementary Table 4** Patient baseline characteristics in group HP

|  | | AC-T | TCb | T | P-value |
| --- | --- | --- | --- | --- | --- |
|  | | N=36(%) | N=14(%) | N=5(%) |  |
| Age | | | | | |
|  | ≤50 | 24(66.7) | 7(50.00) | 3(60.00) | 0.572 |
|  | ＞50 | 12(33.3) | 7(50.00) | 2(40.00) |  |
| Menopausal status | | | | | |
|  | Pre | 21(58.33) | 8(57.14) | 3(60.00) | 1.000 |
|  | Post | 15(41.67) | 6(42.86) | 2(40.00) |  |
| cT stage (pre-treatment) | | | | | |
|  | 1 | 4(11.11) | 0(0) | 1(20.00) | 0.180 |
|  | 2 | 27(75.00) | 10(71.43) | 2(40.00) |  |
|  | 3 | 1(2.78) | 2(14.29) | 1(20.00) |  |
|  | 4 | 411.11) | 2(14.29) | 1(20.00) |  |
| HR | | | | | |
|  | Negative | 15(41.67) | 10(71.43) | 2(40.00) | 0.170 |
|  | Positive | 21(58.33) | 4(28.570 | 3(60.00) |  |
| HER2 | | | | | |
|  | IHC 2+/FISH+ | 3(8.33) | 2(14.29) | 0(0) | 0.763 |
|  | IHC 3+ | 33(91.67) | 12(85.71) | 5(100) |  |
| Ki67 | | | | | |
|  | <20% | 3(8.33) | 3(21.43) | 1(20.00) | 0.329 |
|  | ≥20% | 33(91.67) | 11(78.57) | 4(20.00) |  |
