# Supplementary material for: PR status is a more decisive factor in efficacy of adding pertuzumab into neoadjuvant therapy for HER2-positive and lymph node-positive breast cancer than ER status: a real-world retrospective study in China
Source: World J Surg Oncol. 2023 Sep 18;21:296. doi: 10.1186/s12957-023-03178-4 (PMC10506239; doi:10.1186/s12957-023-03178-4)
Supplement: Supplementary file 5 — Additional file 5: Supplementary Table 5. Characteristics of patients achieved pCR in group HP. [file 12957_2023_3178_MOESM5_ESM.docx]

**Supplementary Table 5** Characteristics of patients achieved pCR in group HP

| Characteristics | | AC-T | | | TCb | | | T |
| --- | --- | --- | --- | --- | --- | --- | --- | --- |
|  |  | N(%) | Exp(B)(95%CI) | P-value | N(%) | Exp(B)(95%CI) | P-value | N(%) |
| Total | | 21(58.33) |  |  | 9(64.29) |  |  | 2(40.00) |
| Age | | | | | | | | |
|  | ≤50 | 14(58.33) | 1.000(0.245-4.078) | 1.000 | 4(57.14) | 0.533(0.058-4.912) | 0.579 | 1(33.33) |
|  | ＞50 | 7(58.33) | Reference |  | 5(71.43) | Reference |  | 1(50.00) |
| Menopausal status | | | | | | | | |
|  | Pre | 12(57.14) | 0.889(0.231-3.418) | 0.864 | 5(62.50) | 0.833(0.090-7.675) | 0.872 | 1(33.33) |
|  | Post | 9(60.00) | Reference |  | 4(66.67) | Reference |  | 1(50.00) |
| cT stage (pre-treatment) | | | | | | | | |
|  | 1-3 | 19(59.38) | 1.462(0.182-11.735) | 0.721 | 9(100) | N/A |  | 2(50.00) |
|  | 4 | 2(50.00) | Reference |  | 0(0) |  |  | 0(0) |
| HR | | | | | | | | |
|  | Negative | 13(86.67) | 10.562(1.873-59.562) | 0.008 | 6(60.00) | 1.333(0.139-12.818) | 0.803 | 2(100) |
|  | Positive | 8(38.10) | Reference |  | 3(75.00) | Reference |  | 0(0) |
| ER | | | | | | | | |
|  | Negative | 16(72.73) | 4.800(1.737-20.272) | 0.033 | 6(60.00) | 0.750(0.078-7.210) | 0.803 | 2(66.67) |
|  | Positive | 5(35.71) | Reference |  | 3(75.00) | Reference |  | 0(0) |
| PR | | | | | | | | |
|  | Negative | 16(88.89) | 10.000(2.059-48.558) | 0.004 | 8(72.73) | 5.333(0.343-82.827) | 0.233 | 2(100) |
|  | Positive | 5(27.78) | Reference |  | 1(33.33) | Reference |  | 0(0) |
| HER2 | | | | | | | | |
|  | IHC 2+/FISH+ | 0(0) | N/A | 0.999 | 1(50.00) | 0.500(0.024-10.251) | 0.653 | 0(0) |
|  | IHC 3+ | 21(100) |  |  | 8(66.67) | Reference |  | 2(40.00) |
| Ki67 | | | | | | | | |
|  | <20% | 2(66.7) | 1.474(0.121-17.913) | 0.761 | 2(66.67) | 1.143(0.077-16.947) | 0.923 | 0(0) |
|  | ≥20% | 19(57.58) | Reference |  | 7(63.64) | Reference |  | 2(50.00) |
