# Supplementary material for: PR status is a more decisive factor in efficacy of adding pertuzumab into neoadjuvant therapy for HER2-positive and lymph node-positive breast cancer than ER status: a real-world retrospective study in China
Source: World J Surg Oncol. 2023 Sep 18;21:296. doi: 10.1186/s12957-023-03178-4 (PMC10506239; doi:10.1186/s12957-023-03178-4)
Supplement: Supplementary file 6 — Additional file 6: Supplymentary Figure. a.Patients with different chemotherapy regimens in group HP; b. Patients treated with AC-T regimen with different PR status in group HP. [file 12957_2023_3178_MOESM6_ESM.docx]

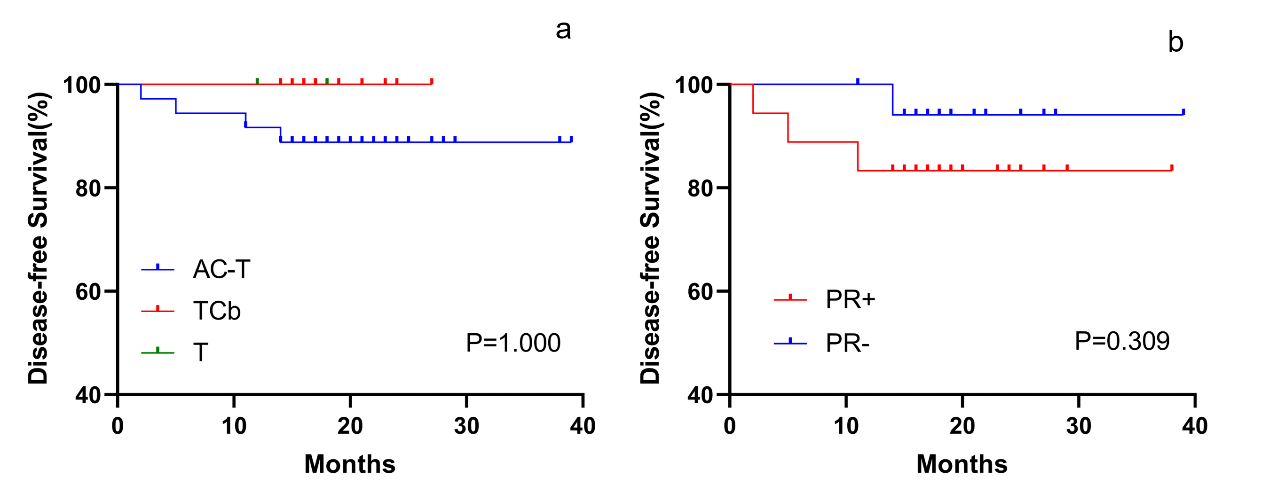


**Supplymentary Figure**

a. Patients with different chemotherapy regimens in group HP; b. Patients treated with AC-T regimen with different PR status in group HP
